# Conformational Changes Induced by S34Y and R98C Variants in the Death Domain of Myd88

**DOI:** 10.3389/fmolb.2020.00027

**Published:** 2020-03-24

**Authors:** Vijayakumar Gosu, KyeongHye Won, Jae-Don Oh, Donghyun Shin

**Affiliations:** ^1^Department of Animal Biotechnology, Jeonbuk National University, Jeonju-si, South Korea; ^2^The Animal Molecular Genetics and Breeding Center, Jeonbuk National University, Jeonju-si, South Korea

**Keywords:** Myd88, polymorphism, molecular dynamics simulation, principal components, betweenness centrality

## Abstract

Myeloid differentiating factor 88 (Myd88) is a universal adaptor protein that plays a critical role in innate immunity by mediating TLR downstream signaling. Myd88 death domain (DD) forms an oligomeric complex by association with other DD-containing proteins such as IRAK4. Despite its universal role, polymorphisms in Myd88 can result in several diseases. Previous studies have suggested that, out of several non-synonymous single-nucleotide polymorphisms (nsSNPs), the variants S34Y and R98C in the DD of Myd88 disrupt the formation of the Myddosome complex. Therefore, we performed molecular dynamics (MD) simulations on wild-type (Myd88^WT^) and mutant (Myd88^S34Y^, Myd88^R98C^) DDs to evaluate the subtle conformational changes induced by these mutations. Our results suggest that the S34Y variant induces large structural transitions compared to the R98C variant as evidenced by residual flexibility at the variable loop regions, particularly in the H1–H2 loop, and variations in the collective modes of motion observed for wild-type and mutant Myd88 DDs. The residue interaction network strongly suggests a distortion in the interaction pattern at the location of the mutated residue between the wild type and mutants. Moreover, betweenness centrality values indicate that variations in the distribution of functionally important residues may be reflected by distinct residue signal transductions in both wild-type and mutant Myd88 DDs, which may influence the interaction with other DDs in TLR downstream signaling.

## Introduction

Pattern recognition receptors (PRRs) play a crucial role in triggering the host innate immune response against harmful microbial organisms (Takeuchi and Akira, 2010; Thompson et al., 2011). Toll-like receptors (TLRs) are an important class of PRRs that are activated when pathogen-associated molecular patterns (PAMPs) are sensed. There are about 13 TLRs in mammals, and each TLR senses PAMPs with different specificities. Few TLRs, such as TLR3, TLR7/8, and TLR9, are localized in endosomes, whereas other TLRs are localized extracellularly. Intracellular TLRs recognize nucleic acids, whereas extracellular TLRs such as TLR2, TLR4, and TLR5 recognize lipopeptide (TLR2), lipopolysaccharide (LPS), and flagellin, respectively (Kawai and Akira, 2010; Kawasaki and Kawai, 2014). When PAMPs are detected, TLRs trigger the signaling mediators and activate NF-κB, thereby inducing proinflammatory genes encoding cytokines and chemokines (Gosu et al., 2012; Liu et al., 2017). TLR signaling is predominantly described as myeloid differentiating factor 88 (Myd88)-dependent and Myd88-independent pathways. Myd88 is a universal adaptor protein that associates with all TLRs except TLR3. TLR3 is mediated through adaptor proteins such as TRIF. In humans, there are five adaptor proteins such as Myd88, TRIF, Mal, TRAM, and SARM in TLR signaling (Troutman et al., 2012). Out of these, MyD88 seems to be crucial because of its universal role in signaling and involvement in innate immunity. Myd88-deficient mice have been shown to be susceptible to leishmanial infection (Muraille et al., 2003; von Bernuth et al., 2012), and Myd88-deficient macrophages have been shown to be defective in the production of TNF and NO upon mycobacterial stimulation (Fremond et al., 2004). Moreover, Myd88 point mutations have been associated with several deadly bacterial infections (von Bernuth et al., 2008; Cervantes, 2017). In addition, Myd88 polymorphisms in chicken also increase their susceptibility to salmonella pullorum infection (Liu et al., 2015). The point mutations in Myd88 have been reported in a previous report (von Bernuth et al., 2008). Experimental reports suggest that out of several non-synonymous single-nucleotide polymorphisms (nsSNPs), S34Y and R98C variants interfere with the Myddosome complex (George et al., 2011). In particular, the S34Y variant is inactive in all Myd88-dependent signaling (Nagpal et al., 2011).

**Figure 1:**
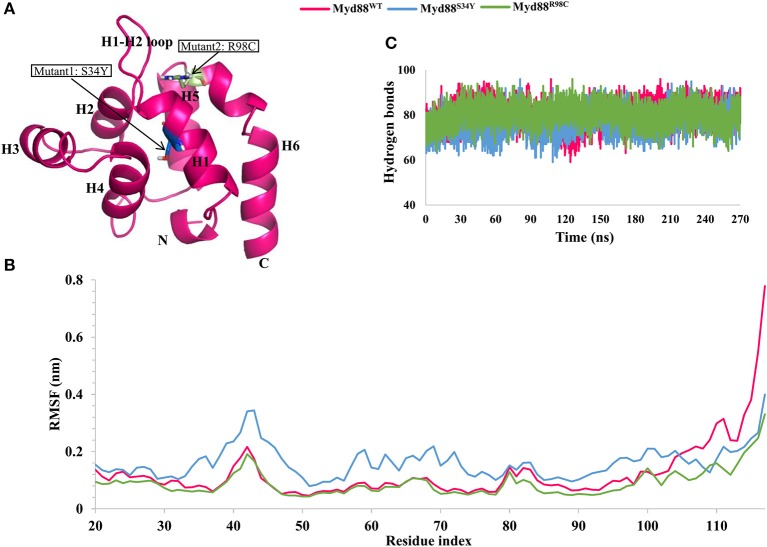
Root mean square fluctuation (RMSF) and hydrogen bond analysis. **(A)** The structure of Myd88 DD. Mutant positions are highlighted. **(B)** The RMSF of backbone atoms of concatenated trajectory (270 ns) from all replicates of each system for wild-type and mutant Myd88. **(C)** The intramolecular hydrogen bonds for concatenated trajectory (270 ns) of wild-type and mutant Myd88.

The Myd88 adaptor protein is 296 amino acids (aa) long and comprises a modular domain structure with N-terminal death domain (DD), intermediate domain (ID), and C-terminal TIR domain. The DD is crucial in forming the Myddosome complex via interactions with IRAK4 and IRAK1/IRAK2 DDs, whereas the TIR domain is important for initiating downstream signaling via interactions with the TLR-TIR domain. Extensive structural analyses have been performed on the TIR domains, which is largely conserved among adaptor proteins as well as TLRs (Ohnishi et al., 2009; Mahita and Sowdhamini, 2018). However, there have been very few studies on the structure of Myd88 DD and the variants of Myd88. The Myddosome complex has been resolved through protein crystallography, which suggested that the Myd88 DD is oligomeric in solution with six molecules of Myd88, four molecules of IRAK4, and four molecules of IRAK2. The DD is a small domain that is ~90 aa, and is composed of six helices connected by loops (Lin et al., 2010) (Figure 1A). The rare point mutations S34Y and R98C have been suggested in a previous report to disrupt the formation of the Myddosome complex (George et al., 2011; Nagpal et al., 2011). Moreover, several studies have been reported that show that perturbations due to point mutations originate allosteric mechanism of protein functional activity (Guarnera and Berezovsky, 2019a,b; Tan et al., 2019; Tee et al., 2019). Hence, studies on conformational changes or allosteric mechanism induced by point mutations in the DD of Myd88 may provide structural insights to better understand how the single point mutations influence the DD–DD interactions.

In this study, we evaluated the wild-type Myd88 DD (Myd88^WT^) and Myd88 mutants (Myd88^S34Y^, Myd88^R98C^) using molecular dynamics (MD) simulations of 100 ns with three replicates using different initial velocities. Furthermore, we performed principal component analysis (essential dynamics) and residue network analysis to better understand the subtle conformational changes induced by these point mutations in the DD of Myd88.

## Materials and Methods

### Preparation of Wild-Type and Mutant Structures of Myd88 DD

The Myd88 crystal structure has been previously deposited in the Protein Data Bank (PDB) as a Myddosome complex (Lin et al., 2010). We downloaded the Myddosome complex (PDB ID: 3MOP) and extracted the structure of the Myd88 DD. After initial minimization, we truncated both the N- and C-termini because of the long loop structures and considered the residues between 20 and 117 aa; this was considered the wild-type model (Myd88^WT^). Furthermore, we changed serine to tyrosine at residue 34, and changed arginine to cysteine at residue 98 using the mutation option in PyMOL with probable rotamers to construct mutant models (Myd88^S34Y^ and Myd88^R98C^) of the Myd88 DD. Finally, we assessed the stereochemical properties using the ProQ webserver.

### MD Simulation Protocol

We subjected all of the structures (Myd88^WT^, Myd88^S34Y^, and Myd88^R98C^) to atomistic MD simulations using Gromacs 5.1.4 (Van Der Spoel et al., 2005; Pronk et al., 2013) with AMBER-ff99SB-ILDN force field (Lindorff-Larsen et al., 2010) similar to our previous report (Gosu et al., 2019). At first, we prepared the topology files using pdb2gmx, placed the structure in a dodecahedral box, and maintained a periodic distance of 12 nm from the protein to the box wall. Tip3p water molecules 6,947, 6,945, and 6,947 were included for Myd88^WT^, Myd88^S34Y^, and Myd88^R98C^, respectively. To neutralize the system, four, four, and five sodium ions were included for Myd88^WT^, Myd88^S34Y^, and Myd88^R98C^, respectively. The energy minimization was performed using the steepest descent method with 1,000 kJ/mol/nm as a maximum tolerance. Using the energy-minimized structure, NVT equilibration simulations were performed for 100 ps. Subsequently, an NPT equilibration simulation was performed for 500 ps using positional restraints. All of the bonds were restrained using the Lincs algorithm, and short-range electrostatics and Van der Waals interactions were accounted for with a 1.0-nm cutoff, and long-range electrostatics were maintained using the Particle Mesh Ewald (PME) method. Temperature (300 K) and pressure (1.0 bar) were maintained using v-rescale, a modified Berendsen thermostat, and Parrinello-Rahman barostat, respectively. Production simulations were performed without positional restraints for 100 ns. Three independent replicate simulations using different initial velocities were performed for all the systems.

### Principal Components (PCs) and Free Energy Landscape (FEL) Analysis

PCs calculations were used to obtain concerted motions during simulations that are likely significant for biological function. We calculated PCs on the concatenated trajectory (last 90 ns of each replicate from three systems) as reported in previous reports (Gosu and Choi, 2014; Gosu et al., 2019). After removing the rotational and translational motions, the covariance matrix was constructed and diagonalized, which yielded a set of eigenvalues (amplitude) and eigenvectors (direction of motion). We obtained eigenvalues and eigenvectors using the gmx covar and analyzed the data using the gmx anaeig tool from the gromacs package (van Aalten et al., 1995; Yamaguchi et al., 1998). The gmx sham tool was used to construct the FEL, which is a combination of data points from the reaction coordinates of PC1 and PC2, and plots were drawn using the Mathematica version 12.

### Residue Interaction Networks

Residue interaction networks (RINs) are widely used to understand the impact of mutations on proteins. We constructed representative structures of each system (both wild type and mutants) as a network, in which the nodes represent residues and the edge represents contacts between residues using RINalyzer (Doncheva et al., 2011) and the structure viz module implemented in cytoscape 3.14, similar to a previous report (Anwar and Choi, 2017). The contact distance between any two residues was considered at 5 Å. Furthermore, the network topological parameters were calculated as an undirected network using the network analyzer (Assenov et al., 2008). Betweenness centrality (C_B_), closeness and node degree distribution were calculated. C_B_ is an important factor that suggests the residues crucial for the functional importance of the proteins (Lee et al., 2014; Basith et al., 2019). In addition, we also constructed RIN using the RING2.0 webserver, which was useful to inspect the various interactions such as hydrogen bonds, Van der Waals interactions, and ionic interactions in protein models (Piovesan et al., 2016).

### Evaluation of Allosteric Effects

To quantify the allosteric effects under mutation for Myd88 DD, a statistical mechanical model implemented in the AlloSigMA server (Guarnera et al., 2017) was used. This method estimates the free energy of each residue used by the allosteric communication under mutation or binding events, which is effectively verified in previous studies (Takeuchi and Akira, 2010; Guarnera and Berezovsky, 2016; Su et al., 2018a,b). We initiated perturbations at different positions (34 and 98) using UP mutation to simulate the bulky residues at these positions and subsequently calculate the free energies accountable for allosteric communication.

## Results and Discussion

### Structural Dynamics of Wild-Type and Mutant Myd88 DDs

The Myd88 DD structure (Myd88^WT^) was extracted from the Myddosome complex (PDB ID: 3MOP). After initial inspection, we constructed the mutant Myd88 DD models (Myd88^S34Y^ and Myd88^R98C^). Subsequently, we subjected all three models (Myd88^WT^, Myd88^S34Y^, and Myd88^R98C^) to conventional MD simulations for 100 ns with three independent replicates (total 300 ns) using different initial velocities. To assess the stability of all of the systems, we calculated the root mean square deviation (RMSD) of backbone atoms with respect to the initial structure for the whole MD trajectories. RMSD suggested that all of the replicates showed a consistent increase in RMSD until ~10 ns. Thereafter, RMSD values ranged from 0.1 to 0.3 nm for Myd88^WT^, 0.1–0.4 nm for Myd88^S34Y^, and 0.1–0.2 nm for Myd88^R98C^ (Figure S1). However, the replicates of each system had slight variations in the RMSD values. In particular, Myd88^WT^ showed more variations among replicates. Myd88^WT^ was stable during the whole MD trajectory; however, it showed large flexibility at the end of the simulation (Figure S1). The observed RMSD values between wild-type and mutant models of the Myd88 DD indicated that the global structural deviation did not vary largely. Furthermore, the radius of gyration (Rg) of the backbone atoms revealed that all of the systems were compact during the simulations (Figure S1). However, the difference in Rg values indicate that S34Y mutant in Myd88 may exhibit large conformational changes compared to R98C mutant. In addition, we also observed that solvent accessible surface area (SASA) averaged from three replicates were 61.52, 63.03, and 61.8 for Myd88^WT^, Myd88^S34Y^, and Myd88^R98C^, respectively. Despite the compact folding of the DD, the increase in SASA for mutant Myd88 (in particular Myd88^S34Y^) compared to wild-type Myd88 indicated obvious conformational changes induced by mutations (Figure S1).

### Residue Flexibility and Intra-Hydrogen Bonds During Simulations

From the three replicates of each system, we concatenated the last 90 ns trajectory (a total of 270 ns for each system) for further analysis. We checked residual flexibility using root mean square fluctuations (RMSFs) of backbone atoms for the concatenated trajectory. The variation in the RMSF values suggested that the residual flexibility was similar between the wild-type and mutant systems except at the C-terminal residues and variable loop regions (Figure 1B). Deeper inspection of RMSF values indicated high residue flexibility at the H1–H2 loop region (39–48) in Myd88^S34Y^ compared to both Myd88^WT^ and Myd88^R98C^. Moreover, the residue movements were larger in Myd88^S34Y^ compared to other systems. This was reasonable because the change in serine (small amino acid) to tyrosine (bulky amino acid) at residue 34 may induce conformational changes in the surrounding region (Figure S2). Additionally, Myd88^R98C^ and Myd88^WT^ show similar residue fluctuations; however, large fluctuations for wild type were observed in the region of helix 6 (100–117). Furthermore, we analyzed the intra-hydrogen bonds for all systems, which indicated their slight variations, particularly for the Myd88^S34Y^ mutant. On average, 80, 77, and 80 hydrogen bonds were observed for Myd88^WT^, Myd88^S34Y^, and Myd88^R98C^, respectively (Figure 1C). The above analyses cumulatively suggested that mutations in Myd88 DD had an impact on the overall structural organization, which may influence the DD–DD interaction in the downstream signaling of TLRs.

### Collective Motions of Wild-Type and Mutant Myd88 DD

**Figure 2:**
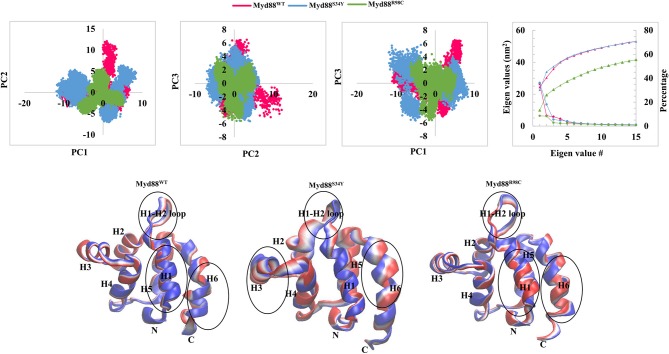
Collective modes of motions. **(Top panel)** The projection of PC1 and PC2, PC2 and PC3, and PC1 and PC3 is shown. The covariance of each eigenvalue with respective eigenvector is shown. In addition, cumulative percentage is also shown. **(Bottom panel)** Two extreme conformations with 30 frames from the concatenated trajectory for PC1 are shown and sequentially superimposed. The minimum (red) to maximum (blue) conformations are shown. The circle represents the large conformational changes.

In order to assess the dominant modes and conformational changes particularly induced by mutations, we performed a principal component analysis (PCA) on the concatenated trajectory (270 ns) of each system. The PCA indicated that the first few eigenvectors had eigenvalues >1 nm^2^ as shown in Figure 2. The diagonalized co-variances of wild-type and mutant models were 81.19 for Myd88^WT^, 89.9 for Myd88^S34Y^, and 48.65 for Myd88^R98C^. This indicated that Myd88^S34Y^ underwent a large fluctuation compared to Myd88^WT^ and Myd88^R98C^. The cumulative percentage of mean square fluctuation for the first 15 eigenvectors were 68% for Myd88^WT^, 69% for Myd88^S34Y^, and 55% for Myd88^R98C^. Furthermore, the first three eigenvectors contribute large motions, i.e., 60% for Myd88^WT^, 61% for Myd88^S34Y^, and 32% for Myd88^R98C^ (Figure 2). To assess the possible reason for less global dynamics (only 32% for first three PCs), of the Myd88^R98C^ mutant, we performed the RMSD calculations at residue 98 position for concatenated trajectory of all models, which show that Arg at the 98 position is largely flexible compared to Cys at position 98 in mutant (Myd88^R98C^) model (Figure S2A). The minimum distance calculations between Arg98/Cys98 with Phe36 from helix 1, Ala45 from the H1–H2 loop, and Asp100 from helix 6 suggest possible local structural alterations; in particular, the minimum distance between Cys98 and Ala45 from the H1–H2 loop is higher in Myd88^R98C^ compared to wild type (Figure S2C). Moreover, the SASA (Figure S2E) and atomic fluctuations (Figure S3A) at the 98 position is largely varied compared to wild type. All the above analysis suggests that this mutant (R98C) may alter the interaction pattern particularly with the H1–H2 loop at the mutant surrounded region, thereby leading to less dynamics globally compared to Myd88^WT^ and Myd88^S34Y^. Additionally, the trajectories obtained for the first three PCs were projected on the phase space (Figure 2), which indicated that the Myd88^S34Y^ mutant was more dynamic compared to other systems. The spreading of the trajectory on the phase space clearly indicated few clusters for the Myd88^S34Y^ mutant, whereas Myd88^R98C^ shows less spreading of the trajectory compared to wild type. Hence, it was evident that the serine-to-tyrosine mutation at residue 34 had a large impact on the structure within the Myd88 DD.

**Figure 3:**
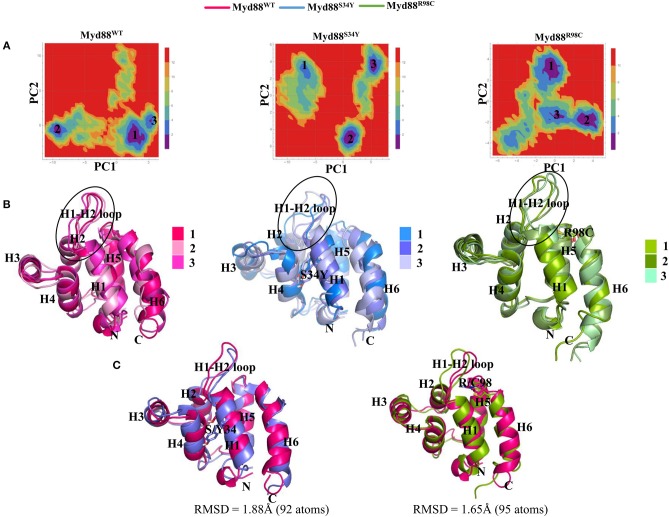
Free energy landscape (FEL) and representative structures. **(A)** FEL of the wild-type and mutant Myd88 are shown using PC1 and PC2 as reaction coordinates. **(B)** The representative structures of Myd88 were considered and superimposed. The black circle represents the variation in the H1–H2 loop. **(C)** The superimpositions of the wild-type representative structure on both mutants are shown along with RMSD values.

In order to assess the variations in the collective motions of the wild-type and mutant systems, we considered two extreme positions with 30 frames form the concatenated trajectory of all the systems along the first three eigenvectors as shown in Figure 2 and Figure S3B. In addition, Movies S1–S9 show animations of the motions for all systems along the first three eigenvectors. The mutant Myd88, in particular Myd88^S34Y^, shows overall large fluctuations. The first dominant mode (PC1) in Myd88^WT^ shows that the H1–H2 loop region (39–48) moves toward helix 3; in contrast, the mutants moved in the opposite direction (Figure 2 and Movies S1–S3). However, large motions were observed in Myd88^S34Y^, indicating that this mutant altered the structural organization of Myd88 DD, which may influence the symmetry required for Myddosome formation. Moreover, helix 6 was observed to undergo large motion in both the wild type and mutants. The second and third dominant modes (PC2 and PC3) show overall large motion in helix 6 as well as in the H1–H2 loop (Figure S3B and Movies S4–S9). Furthermore, in order to assess the low-energy structures, we performed a FEL analysis, which suggested that Myd88^WT^ may not undergo large conformations during simulations compared to mutants. Importantly, Myd88^S34Y^ underwent large conformations as shown in Figures 3A,B, particularly in the H1–H2 loop. Myd88^R98C^ exhibits less conformational difference; however, this mutant also shows variation in the H1–H2 loop region compared to Myd88^WT^ (Figure 3C).

### RINs and Network Centrality

**Figure 4:**
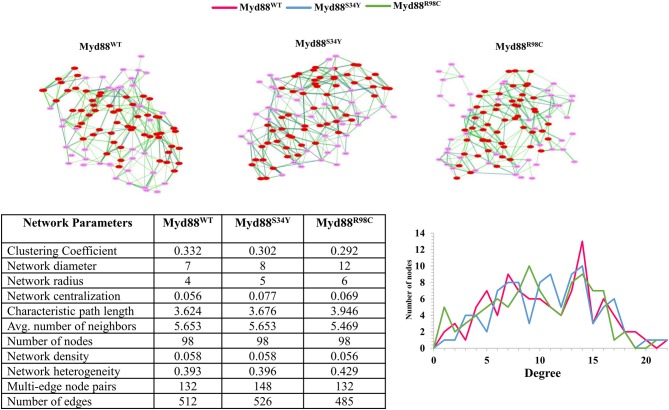
Network topology. **(Top)** The residue networks constructed using a cutoff of 0.5. The residues located in the helices are indicated in red, whereas residues located in the loop are indicated in light pink. **(Bottom)** The residue network properties are shown in the table for wild-type and mutant Myd88. **(Bottom right)** The node degree distribution is shown.

**Figure 5:**
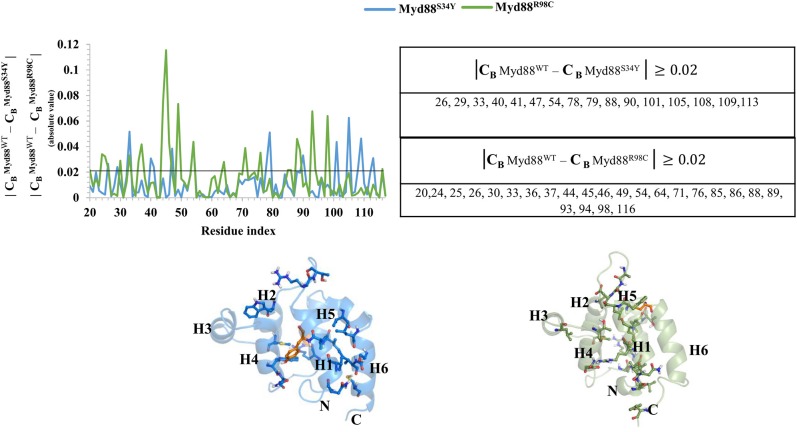
Network centrality. The variation in the betweenness centrality (C_B_) between wild-type and mutant Myd88 is shown. Residues showing C_B_ ≥ 0.02 are presented in the table. The residues identified are shown on the corresponding structures of Myd88^S34Y^ and Myd88^R98C^. The mutant residues Y34 and C98 are shown in orange color on the corresponding structures.

**Figure 6:**
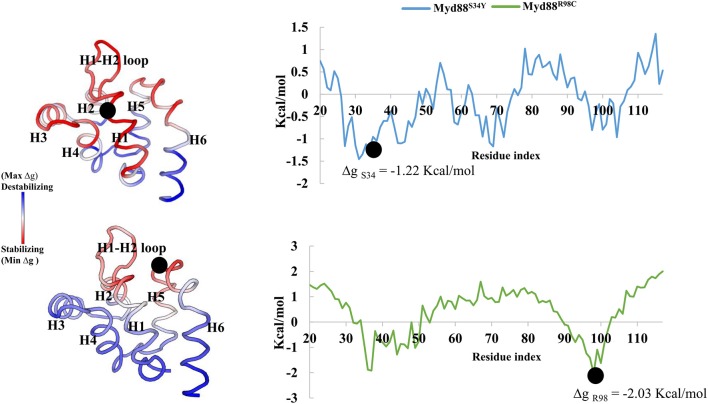
Free energy of Myd88 mutants. Dot represents the mutant residue in the corresponding mutant model.

Recently, it has become common to consider graph theory to construct the RINs, thereby identifying the crucial residues for protein function. To understand the difference in the flow of information for wild-type and mutant Myd88, we constructed RINs on the representative structures using RING (default parameters) and RINlyzer with a contact threshold of 5 Å and then analyzed the topological parameters using the network analyzer. Each residue was considered as node and the contacts between the nodes are considered as edges. The RING analysis, which is useful to understand multiple interaction types involved in the residue networks, indicated that the hydrogen bond and van der Waals interactions largely varied at the location of the mutations, suggesting that this distinct conformation at the mutant position lead to the overall conformational changes in the DD (Figure S4). The RIN and RIN topological parameters are shown in Figure 4. The node degree distribution indicated large variations in RINs between wild-type and mutant Myd88. The network diameter and radius varied among wild-type and mutant Myd88; however, the clustering coefficient and characteristic path length indicated that the network belonged to small world topology. Moreover, the total number of edges was varied among the three systems, indicating that each one exhibits significant differences in signal transduction within the protein (Figure 4). Subsequently, we calculated betweenness centrality (C_B_), closeness (C_C_), and degree (C_D_) for indications of large variations between wild-type and mutant Myd88 (Figure S5). In particular, betweenness centrality was useful for identifying the functionally important residues that are involved in transducing the signal within the protein. Hence, we considered the cutoff (C_B_ ≥ 0.04) and illustrated the residues to better understand the differences in wild-type and mutant Myd88 (Figure S5). We observed that the distribution of functionally important residues varied in the wild type and mutants. For an in-depth understanding, using the condition (|**C**_B_^Myd88^^WT^ – **C**_B_^Myd88S34Y^| ≥ 0.02), (|**C**_B_^Myd88^^WT^ – **C**_B_^Myd88S34Y^| ≥ 0.02), we calculated the betweenness centrality variation between wild-type and mutant models (Figure 5). From this analysis, we observed that some of the residues in both the mutants were similar, which indicated that the mutation in Myd88 DD may lead to an allosteric mechanism. In order to assess the allosteric mechanism under mutation, free energy (Δg_res_) of each residue was calculated using the AlloSigMA server. The prediction of free energy (Δg_res_) indicated that the residues surrounded by the mutant at the 34 position is stabilized (negative free energy); however, the H2–H3 loop, the H4–H5 loop, and helix 5 were destabilized (positive free energy). Similarly, the residue free energy (Δg_res_) calculated under mutation at position 98 indicated less stabilization (negative free energy) at the surrounding region of position 98 and destabilization (positive free energy) in the region of helices 1, 3, 4, and 6 (Figure 6). Compared to S34, R98 mutant shows a large destabilized region, indicating that R98 side chain is involved in interactions between the H1–H2 loop, helix 1, and helix 5; however, upon mutation, these interactions may be disturbed as shown from minimum distance calculations (Figure S2). Hence, it is possible that point mutation in Myd88 DD may exert an allosteric mechanism to regulate the function of protein, which is similar to previous reports suggesting the possible allosteric mechanism of protein function induced by point mutations through perturbations (Guarnera and Berezovsky, 2019a,b; Tee et al., 2019).

## Conclusion

Myd88 is a crucial signaling adaptor in TLR signaling and is associated with the innate immune system. Myd88 deficiency in mice results in several immune diseases. Moreover, the polymorphisms in TLR signaling mediators are important because of the crucial role of these molecules in innate immunity. Hence, in this study, we performed MD simulations on wild-type and mutant Myd88 DDs to better understand the subtle conformational changes induced by the mutations in the DD of Myd88. First, we extracted the crystal structures of the Myd88 DD from PDB (ID: 3MOP) and then applied mutations by replacing serine with tyrosine at position 34 and arginine with cysteine at position 98. Finally, all three models (Myd88^WT^, Myd88^S34Y^, and Myd88^R98C^) were subjected to MD simulations. Our results suggest that the high residue flexibility (in the mutant model Myd88^S34Y^) in the H1–H2 loop region at residues 39–48 may affect Myddosome formation. Furthermore, we observed variations largely at the loop regions of Myd88^S34Y^ compared to other systems (Myd88^WT^ and Myd88^R98C^), which strongly indicated that the S34Y mutation induced large conformational changes particularly at the loop regions and might affect the symmetry required for Myddosome formation. The variation in the betweenness centrality (C_B_) as well as changes in the free energies (Δg_res_) of each residue upon mutation indicate that point mutation in Myd88 DD affects the dynamics, which may lead to allosteric regulation of Myd88 functional activity. MDs and residue network analysis are powerful tools to better understand the structure–function relationship of proteins. We hope that the results obtained from this study may help in understanding allosteric synergism induced by mutations in the DD of Myd88, which may have an influence in the formation of the Myddosome complex.

## Data Availability Statement

The raw data supporting the conclusions of this article will be made available by the authors, without undue reservation, to any qualified researcher.

## Author Contributions

VG designed the work and performed the simulations. VG and KW analyzed the data. VG, J-DO, and DS wrote the paper. J-DO and DS supervised the work. All authors read and approved the manuscript.

### Conflict of Interest

The authors declare that the research was conducted in the absence of any commercial or financial relationships that could be construed as a potential conflict of interest.
