# Supplementary material for: Conformational Changes Induced by S34Y and R98C Variants in the Death Domain of Myd88
Source: Front Mol Biosci. 2020 Mar 24;7:27. doi: 10.3389/fmolb.2020.00027 (PMC7106778; doi:10.3389/fmolb.2020.00027)
Supplement: Supplementary file 1 [file Data_Sheet_1.pdf]

Supplementary Material

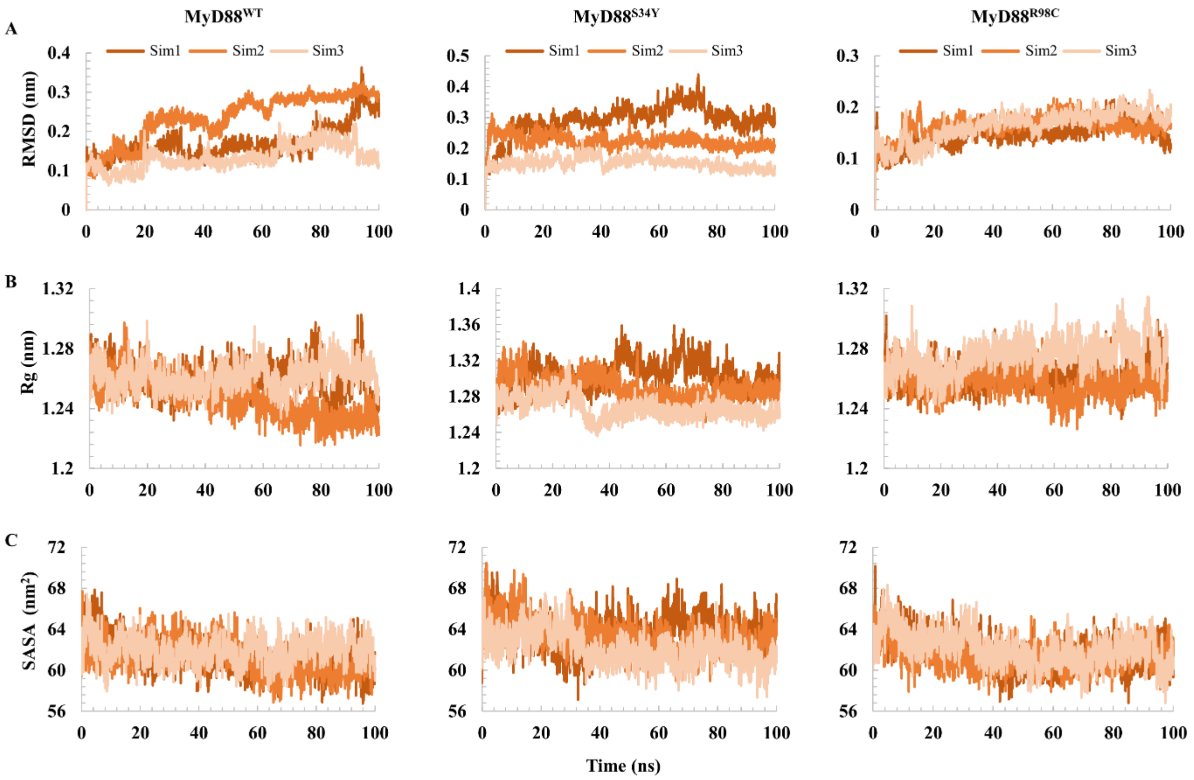

Figure S1. Stability of MD trajectories. (A) The backbone RMSD. (B) Radius of gyration (Rg) of backbone atoms. (C) Solvent accessible surface area (SASA).

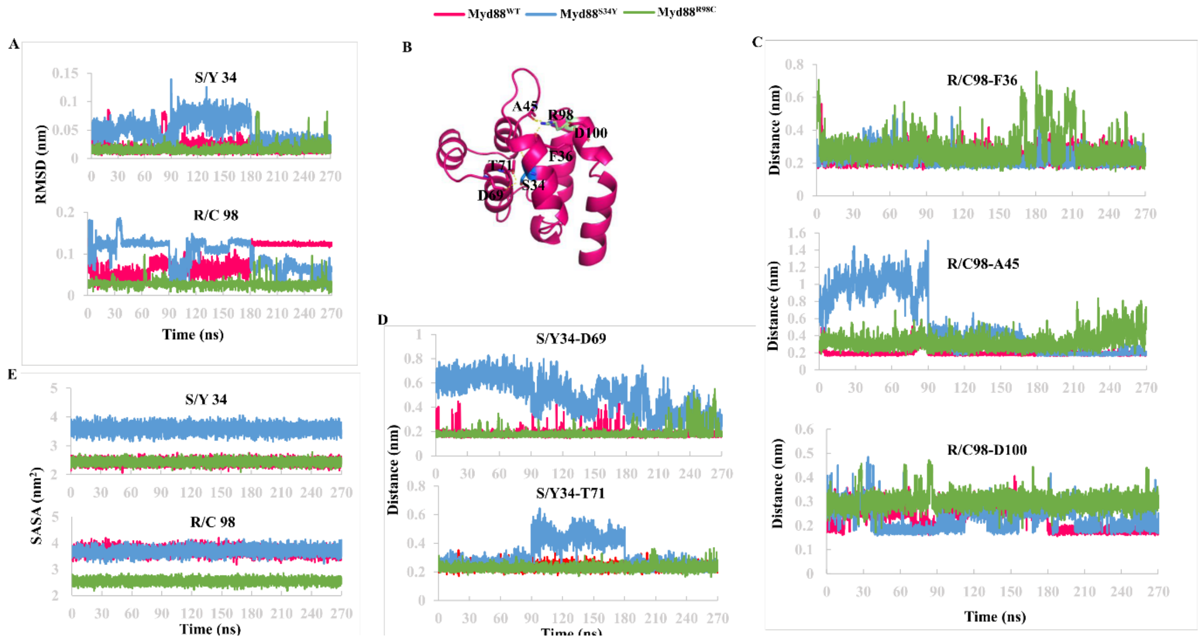

Figure S2. Minimum distance analysis. (A). RMSD of S/Y34 and R/C98 residues. (B) The wild -type structure of Myd88 DD. Mutants S34 (blue) R98 (green) and its interacting residues were shown using stick model. (C) and (D). The minimum distance between interacting residues of S/Y34 and R/C98. All the analysis was done using concatenated trajectory of last 90 ns from 3 replicates (total 270ns). Each 90ns trajectory represent one replicate simulation. (E). The SASA of S/Y34 and R/C98 residues.

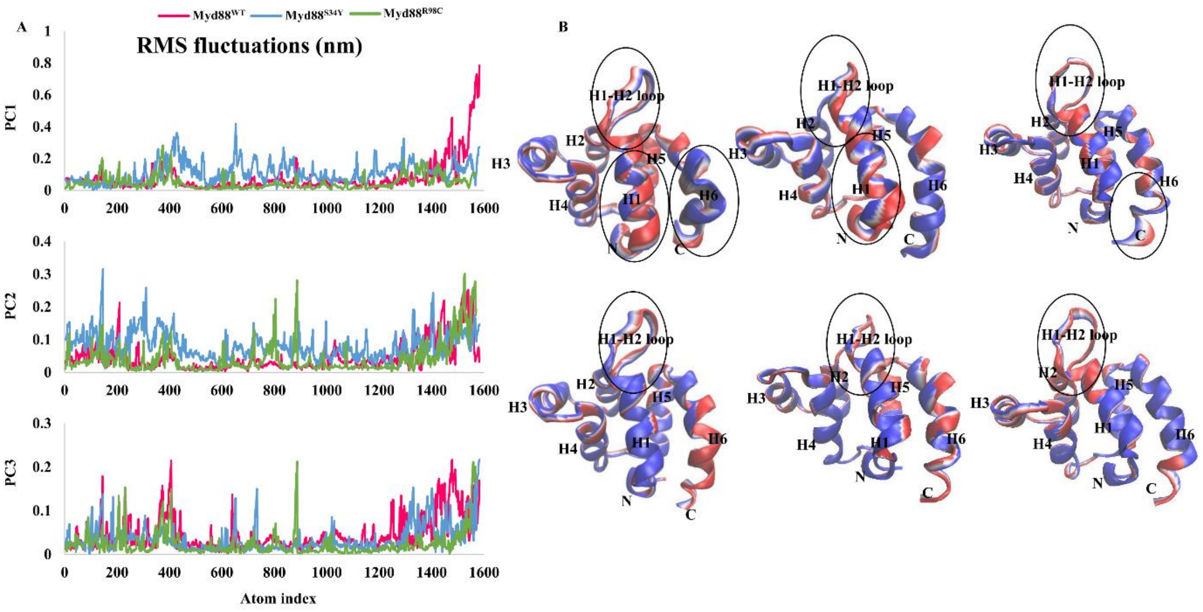

Figure S3. Collective modes of motion. (A). The atomic RMS fluctuations from the concatenated trajectory were shown for PC1 to PC3. (B). Two extreme conformations with 30 frames from the concatenated trajectory for PC2 (top) and PC3 (bottom) are shown and sequentially superimposed. The minimum (red) to maximum (blue) conformations are shown. The circle represents the large conformational changes.

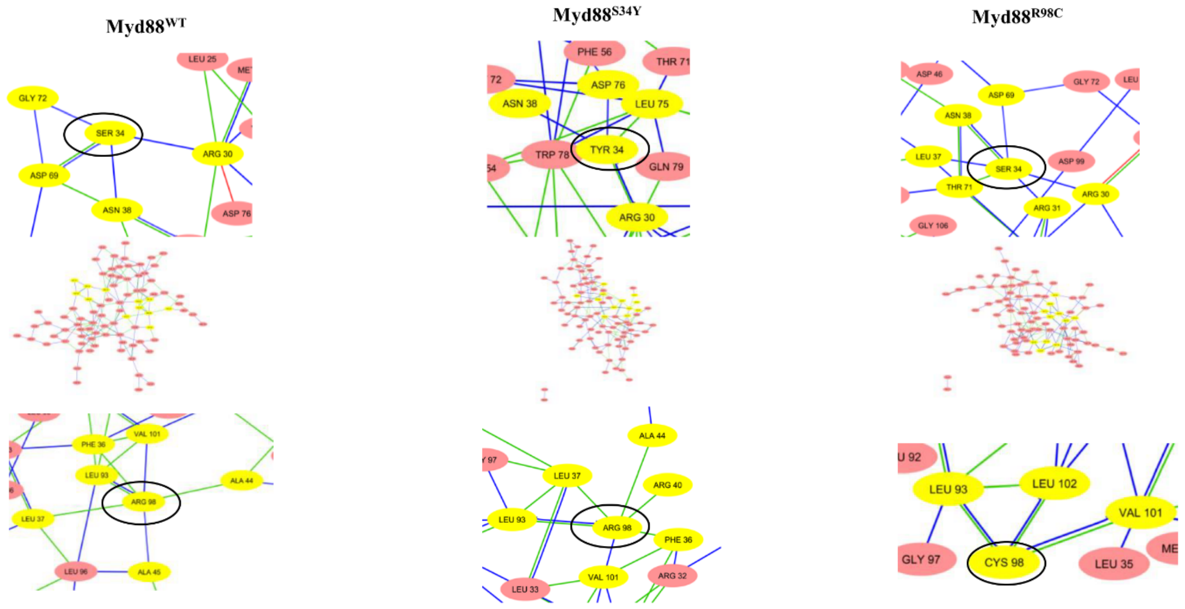

Position	Myd88^{WT}	Myd88^{S34Y}	Myd88^{R98C}
**34**	R30, N38, D69, G72	R30, N38, L75, D76	R30, R31, L37, N38, D69, T71
**98**	F36, L37, A44, A45, L93, V101	F36, L37, R40, A44, L93, V101	L93, V101, L102

Figure S4. Multiple interactions identified through residue networks. The hydrogen bond (blue), Van der Waals interaction (green), and ionic interaction (red) are shown as a network in both wild-type and mutant Myd88. The black circle represents the mutant residues and first neighbor nodes are highlighted using yellow color. The table illustrates the first neighbor nodes at residues 34 (S34Y) and 98 (R98C).

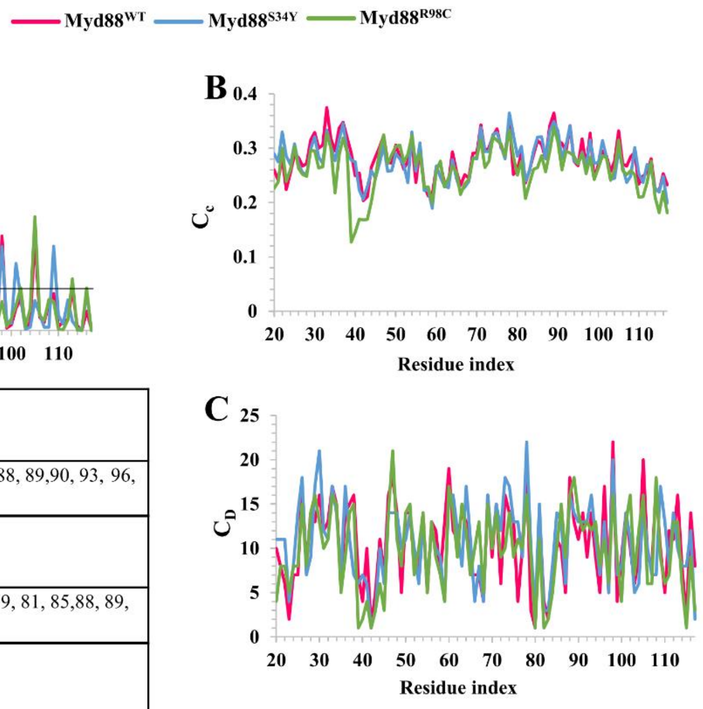

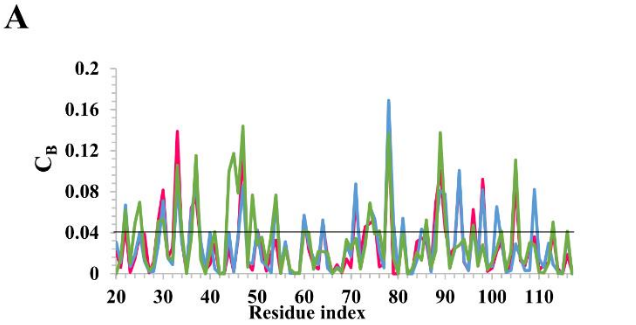

**C_B** Myd88[WT] ≥ 0.04
22, 29, 30, 33, 36, 37, 47, 50, 60, 64,71, 73,74, 75,78, 81,88, 89,90, 93, 96, 98,105
**C_B** Myd88[S34Y] ≥ 0.04
22, 30, 33, 36, 37, 44, 47, 50, 54, 60, 64, 71, 74, 75, 78, 79, 81, 85,88, 89, 90, 93, 96, 98, 101,109
**C_B** Myd88[R98C] ≥ 0.04
22, 24, 25, 29, 30, 33, 34, 37, 41,44, 45, 46, 47, 49, 54, 60, 61, 74, 76, 78, 86, 89, 90, 96, 102, 105, 113,116

Figure S5. Residue network centrality. (A) Betweenness centrality (C_B), (B) closeness (C_c), and (C) degree (C_D) are shown for wild-type and mutant Myd88. Table illustrates the residues having ≥0.04 values of C_B, which may be crucial for the function.

Supplemental movies

Movies S1-S3. The animation movies generated using chimera, with two extreme positions contain 30 frames along PC1 for Myd88^{WT}, Myd88^{S34Y}, and Myd88^{R98C} respectively.

Movies S4-S6. The animation movies generated using chimera, with two extreme positions contain 30 frames along PC2 for Myd88^{WT}, Myd88^{S34Y}, and Myd88^{R98C} respectively.

Movies S7-S9. The animation movies generated using chimera, with two extreme positions contain 30 frames along PC3 for Myd88^{WT}, Myd88^{S34Y}, and Myd88^{R98C} respectively.
